# Integrin-Mediated Macrophage Adhesion Promotes Lymphovascular Dissemination in Breast Cancer

**DOI:** 10.1016/j.celrep.2019.04.076

**Published:** 2019-05-14

**Authors:** Rachel Evans, Fabian Flores-Borja, Sina Nassiri, Elena Miranda, Katherine Lawler, Anita Grigoriadis, James Monypenny, Cheryl Gillet, Julie Owen, Peter Gordon, Victoria Male, Anthony Cheung, Farzana Noor, Paul Barber, Rebecca Marlow, Erika Francesch-Domenech, Gilbert Fruhwirth, Mario Squadrito, Borivoj Vojnovic, Andrew Tutt, Frederic Festy, Michele De Palma, Tony Ng

**Affiliations:** 1 Richard Dimbleby Department of Cancer Research, Randall Division & Division of Cancer Studies, Kings College London, London, UK; 2 Breast Cancer Now Research Unit, King’s College London, Guy’s Hospital, London, UK; 3 Swiss Institute for Experimental Cancer Research (ISREC), School of Life Sciences, École Polytechnique Fédérale de Lausanne (EPFL), Lausanne, Switzerland; 4 Pathology Core Facility, University College London Cancer Institute, London, UK; 5 Institute for Mathematical and Molecular Biomedicine, King’s College London, London, UK; 6 King’s Health Partners Cancer Biobank, King’s College London, London, UK; 7 Research Oncology, Division of Cancer Studies, Guy’s Hospital, King’s College London, London, UK; 8 Division of Imaging Sciences and Biomedical Engineering, King’s College London, London, UK; 9 Department of Oncology, Cancer Research UK and Medical Research Council, Oxford Institute for Radiation Oncology, University of Oxford, UK; 10 Tissue Engineering and Biophotonics, King’s College London, London, UK; 11 UCL Cancer Institute, University College London, London, UK

**Keywords:** lymphovasculature, macrophages, cancer, remodeling, adhesion, contraction, β4 integrin, TGF-β1, RhoA

## Abstract

Lymphatic vasculature is crucial for metastasis in triple-negative breast cancer (TNBC); however, cellular and molecular drivers controlling lymphovascular metastasis are poorly understood. We define a macrophage-dependent signaling cascade that facilitates metastasis through lymphovascular remodeling. TNBC cells instigate mRNA changes in macrophages, resulting in β4 integrin-dependent adhesion to the lymphovasculature. β4 integrin retains macrophages proximal to lymphatic endothelial cells (LECs), where release of TGF-β1 drives LEC contraction via RhoA activation. Macrophages promote gross architectural changes to lymphovasculature by increasing dilation, hyperpermeability, and disorganization. TGF-β1 drives β4 integrin clustering at the macrophage plasma membrane, further promoting macrophage adhesion and demonstrating the dual functionality of TGF-β1 signaling in this context. β4 integrin-expressing macrophages were identified in human breast tumors, and a combination of vascular-remodeling macrophage gene signature and TGF-β signaling scores correlates with metastasis. We postulate that future clinical strategies for patients with TNBC should target crosstalk between β4 integrin and TGF-β1.

## Highlights

• β4 integrin-expressing macrophages release TGF-β1 near breast cancer lymphovasculature
• TGF-β1 drives β4 integrin clustering on macrophages, enhancing macrophage adhesion
• TGF-β1 signals through RhoA to drive to lymphatic endothelial cell contraction
• Lymphatic remodeling signaling cascade facilitates breast cancer metastasis

## Graphical Abstract

**Figure:**
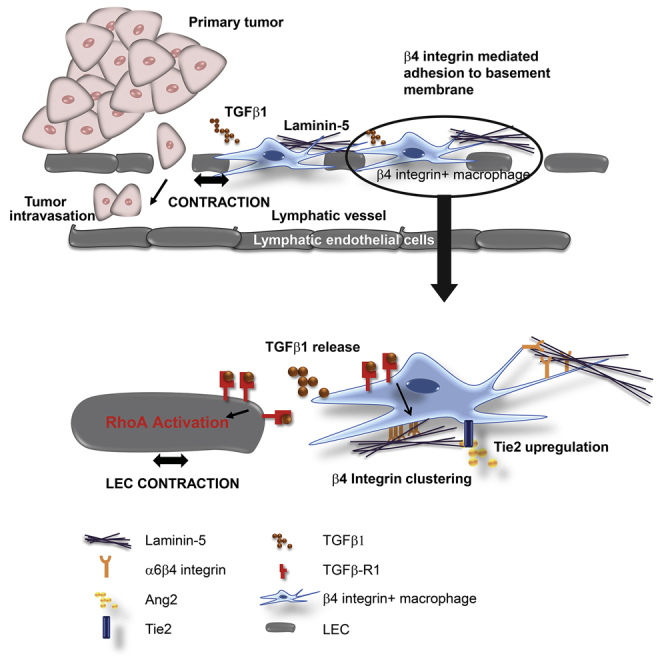

## Teaser

Breast cancer metastasis through lymphatic vessels is associated with poor prognosis. Evans et al. describe β4 integrin-expressing macrophages that regulate lymphatic vessel structure in breast cancer. Macrophage-released TGF-β1 drives lymphatic cell contraction via RhoA activation, culminating in lymphatic hyperpermeability. This study defines a signaling cascade that could be targeted therapeutically.

## Introduction

Tumor cells establish complex interactions with cells within their microenvironment that determine malignancy progression (Balkwill et al., 2012). Tumor cell dissemination can occur through blood or lymphovasculature; however, targeting blood vasculature has limited clinical efficacy when lymphatic dissemination is prevalent (Wong and Hynes, 2006).

Breast cancer is divided into subtypes based on histopathological features and gene signatures (Gazinska et al., 2013).Triple-negative breast cancer (TNBC) is characterized by a lack of druggable targets, is highly metastatic, and is associated with dismal prognosis (Gazinska et al., 2013, Dent et al., 2007). The prognostic significance of lymphangiogenesis in TNBC is under debate. However, invasion into lymphatic vessels correlates with poor prognosis, suggesting that targeting an existing lymphatic vessel network could provide an effective treatment strategy (Choi et al., 2005, Mohammed et al., 2007, Mohammed et al., 2011, Liu et al., 2009).

The relationship between tumor and immune cells is often bidirectional and involves both tumor-promoting and -antagonizing mechanisms (Pollard, 2004, Quail and Joyce, 2013). Among innate immune cells, macrophages have been implicated in the promotion of tumor progression and, in particular, breast cancer metastasis (Condeelis and Pollard, 2006, Kitamura et al., 2015, Pollard, 2004, Harney et al., 2015). However, it remains unclear how certain subsets of tumor-associated macrophages (TAMs) influence breast cancer metastasis spatially, temporally, and at a molecular level.

The integrin family are adhesion receptors of paramount importance for immune cell adhesion and migration during inflammatory processes (Evans et al., 2009). Their ability to form adhesive contacts is regulated by soluble factors, as part of the chemoattractant-adhesion crosstalk that causes a combination of changes in integrin conformation and clustering on the plasma membrane (PM) that regulate downstream signaling (Hynes, 2002). In malignancy, many integrins common in epithelial cells are also present in solid tumors, and some, such as αvβ3 and α5β1, are specifically upregulated in cancer (Desgrosellier and Cheresh, 2010). Tumor-expressed integrins affect tumor cell migration, proliferation, survival, and anchorage to the extracellular matrix. Endothelial-cell-expressed integrins are implicated in angiogenesis, lymphangiogenesis, and vascular remodeling (Avraamides et al., 2008). While the importance of integrins with respect to maintaining a pro-tumoral immune microenvironment in solid tumors is not well defined, in chronic lymphocytic leukemia, impaired integrin signaling in non-leukemic T cells changes the immune microenvironment to be more immunosuppressive, which may facilitate malignancy progression (Ramsay et al., 2013).

We seek to identify the role of TAMs in regulating existing lymphovasculature in TNBC, where lymphatic dissemination is not a direct result of lymphangiogenesis.

We propose that macrophages have an important role in controlling established tumoral lymphatic networks in TNBC and that lymphatic dissemination of cancer cells is facilitated by a cascade of signaling events initiated by integrin-mediated adhesion of macrophages at the sites of lymphatic vessels.

## Results

### Lymphovascular Macrophages in TNBC Mouse Models Are Retained through Binding of β4 Integrin to Laminin-5

**Figure 1:**
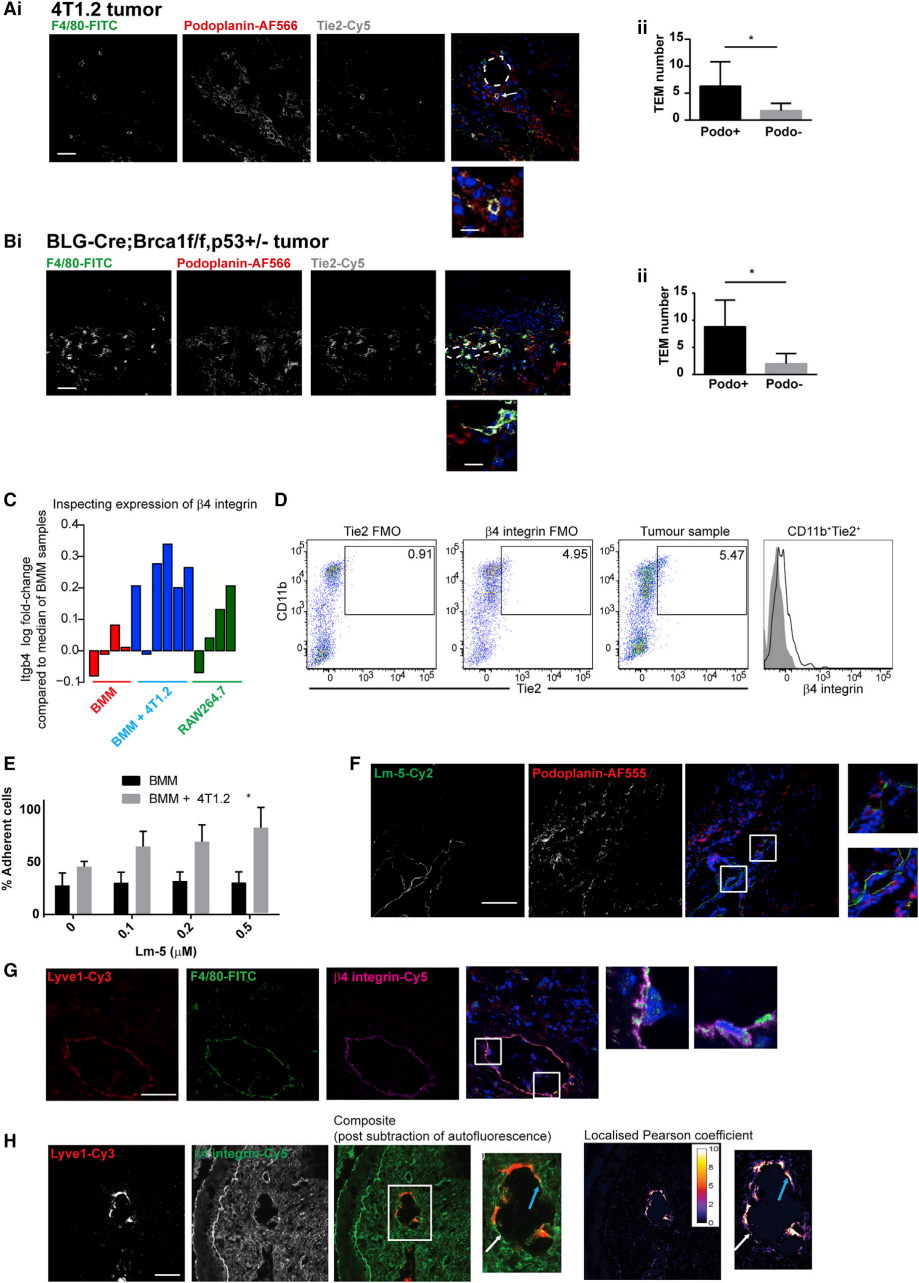
Lymphovascular Macrophages in TNBC Mouse Models Are Retained through Binding of β4 Integrin to Laminin-5 (A and B) Tumor sections from 4T1.2 (A) and BLG-Cre;Brca1^f/f^,p53^+/−^ (B) were stained with F4/80-FITC, podoplanin-AF555, and Tie2 -Cy5-conjugated antibody. F4/80+Tie2+ macrophages within podoplanin+ areas versus those in other areas were quantified per field of view (FOV). Vessel lumen is outlined; arrow indicates a macrophage within a podoplanin+ area. Images were acquired with a ×40 air objective. Scale bars, 100 μm (main image) and 25 μm (zoomed inset). (C) Array-derived expression profile of β4-integrin (Itgb4) across samples. Barplot shows log_2_ fold change of normalized expression value for β4 integrin (ratio of the median value of probe in BMM samples). (D) Day-12 4T1.2 tumors were disaggregated. Tie2 and β4 integrin FMO controls are indicated in 2 left panels. Right dot plot and histogram depict β4-integrin-expressing macrophages from representative 4T1.2 tumor (n = 8). (E) BMMs co-cultured alone or with 4T1.2-GFP cells plated on laminin-5. The percentage of adherent cells were quantified in triplicate (n = 2). (F and G) 4T1.2 tumor sections were stained with laminin-5-Dylight488 and podoplanin-AF555 (F), and Lyve1-Cy3, F4/80-FITC, and β4 integrin-Cy5 (G); inset shows F4/80+β4 integrin+ macrophages around lymphatic endothelium. (H) Stained sections (Lyve1-Cy3 and β4 integrin-Cy5) were imaged using a custom-built microscope (×20 air objective). Area of distinct β4 integrin and Lyve1 within lymphatic vessel (white arrow) and area of close contact between β4 integrin and Lyve1 (blue arrow) are indicated. Scale bars, 50 μm (main panels) and 25 μm (inset).

To identify endogenous macrophages with respect to lymphatic vasculature in murine TNBC tumors, we scored F4/80+Tie2+ macrophages within podoplanin+ lymphovasculature across multiple fields of view (FOVs) from 4T1.2 and BLG-Cre;Brca1^f/f^,p53^+/−^ TNBC models (Molyneux et al., 2010, Melchor et al., 2014; Figures 1A and 1B). The Tie2-expressing macrophage (TEM) subset is associated with angiogenesis and lymphatic development (De Palma et al., 2005, De Palma et al., 2007, Gordon et al., 2010). Lymphovascular-associated macrophages expressing Tie2 have recently been reported in a small breast cancer cohort (Bron et al., 2015). In 4T1.2 tumors, we found a mean value of 6.3 F4/80+Tie2+ macrophages within podoplanin+ vasculature (versus 1.7 in podoplanin− regions) per FOV. In BLG-Cre;Brca1^f/f^,p53^+/−^ tumors, we observed 8.8 F4/80+Tie2+ macrophages in podoplanin+ vasculature (versus 2.0 in podoplanin− regions) per FOV. Therefore, F4/80+Tie2+ macrophages are enriched in lymphovascular regions in murine TNBC models.

The β4 integrin subunit is a transmembrane glycoprotein associating exclusively with the α6 integrin subunit. α6β4 integrin is expressed predominantly on epithelial and endothelial cells and binds to laminins to form adhesion complexes, hemidesmosomes (Stewart and O’Connor, 2015). Microarray analysis of endogenous macrophages co-cultured with 4T1.2 tumor cells showed a mean 1.8-fold upregulation of β4 integrin at the RNA level, compared with non-educated endogenous macrophages, and that the RAW264.7 macrophage cell line similarly exhibited a mean 1.58-fold increase in β4 integrin levels, compared with endogenous macrophages (Figure 1C; see also data published in ArrayExpress: MTAB-4064).

4T1.2 tumors were excised and disaggregated at day 10. Within 4T1.2 tumors, we defined a population of macrophages as CD45^+^Ly6G^−^CD31^−^CD11b^+^Tie2^+^β4 integrin^+^ (Figure 1D).

The influence of tumor education on macrophage adhesion to β4 integrin ligand, laminin-5, was investigated. Tumor-educated endogenous macrophages displayed increased adhesion to laminin-5 (30.7% ± 7.2% to 81.7% ± 13.2% adherent cells on 0.5 μM laminin-5; Figure 1E). As laminin-5 is reportedly localized in areas with high blood vessel density, we investigated whether laminin-5 was also in areas of lymphovasculature. 4T1.2 tumor tissue analysis showed laminin-5 furnished around podoplanin+ lymphovasculature (Figure 1F). In addition we observed macrophages expressing α6β4 integrin in lymphovascular regions (Figure 1G).

To study β4 expression *in vivo*, we used primary 4T1.2 tumor sections stained with Lyve1-Cy3 and β4 integrin-Cy5. Tissues were imaged using a protocol involving laser photobleaching to remove autofluorescence. Our methodology reveals β4 integrin throughout the tumor; however, within lymphatic vessels, there is differential distribution of β4 integrin with relative increases in β4 accumulation observed in lymphovascular areas proximal to Lyve1+ lymphatic endothelial cells (LECs) (Figure 1H, white arrow). Additionally, there were lymphovascular areas with an increased localized Pearson coefficient, suggesting that LECs and β4-integrin-expressing macrophages were in close contact (Figure 1H, blue arrow) (mean colocalization coefficient, 4.094 ± 0.8146).

### TAMs Drive Disorganized and Hyperpermeable Lymphatic Architecture, and Contact between Macrophages and LECs Results in RhoA-Dependent Contraction

**Figure 2:**
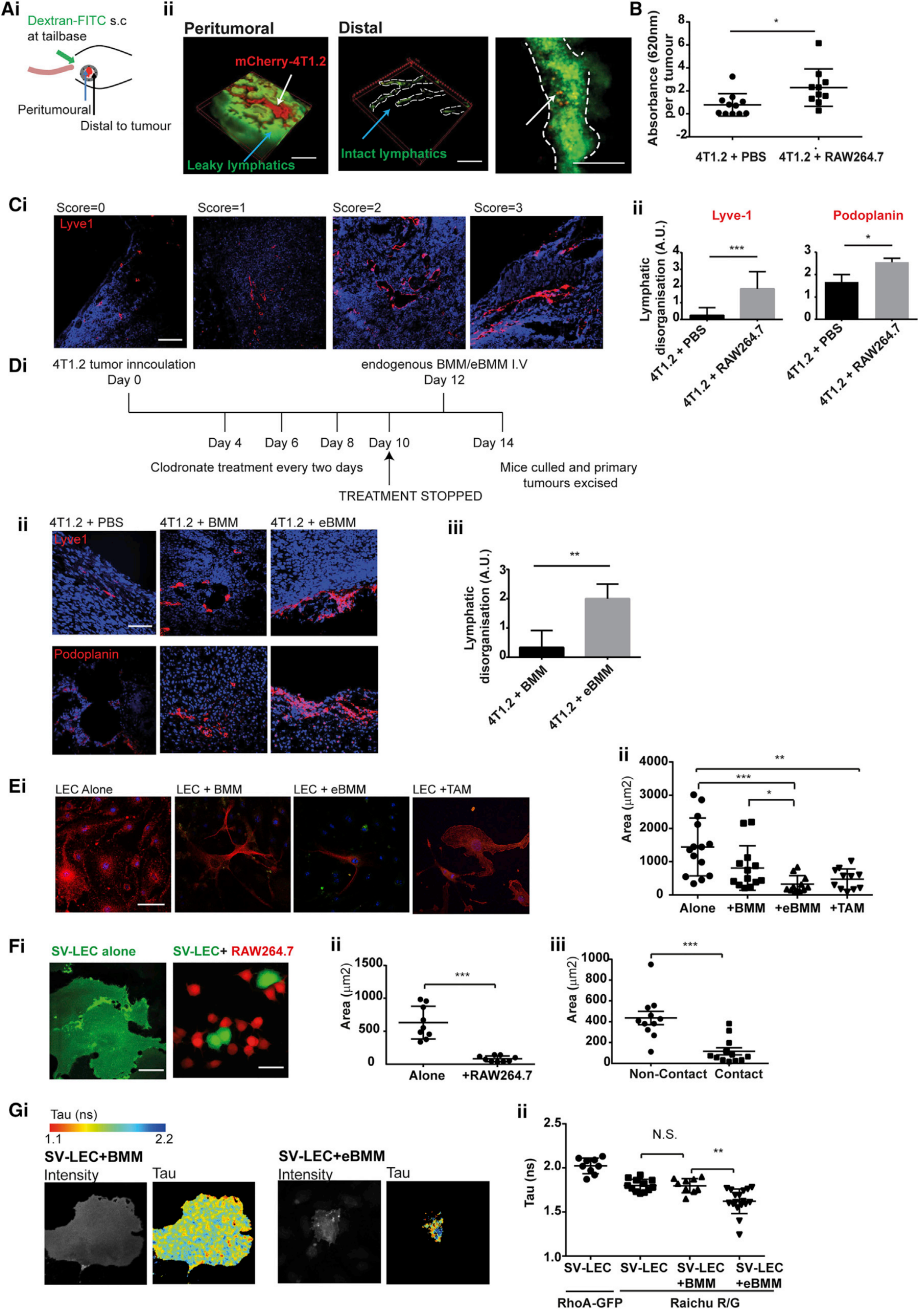
TAMs Drive Dilated, Hyperpermeable, and Disorganized Lymphatic Architecture through LEC RhoA Activation (A) (i) Mouse with mCherry-tagged 4T1.2 tumor and implanted mammary imaging window (MIW) at days 10–14. (ii) Left panel: lymphatic vessels (green) surrounding tumor (red). Middle panel: lymphatic vessels (green) distal to main tumor bulk (red). Right panel: lymphatic vessel (green) with tumor cells (red) within vessel. Scale bars, 100 μm. (B) 4T1.2 tumor-bearing mice were treated with PBS or RAW264.7 macrophages over 3 weeks. 1% Evans Blue dye stained lymphatics *in vivo*. Lymphatic permeability was calculated as optical density per gram of tumor. Data represent means ± SEM; significance was determined using unpaired t tests (^∗∗^p < 0.01). (C) (i) Lymphatic vessels within tumors from mice treated with PBS or RAW264.7 macrophages stained with Lyve1-Cy3 or podoplanin-AF555 (red) and blindly scored for disorganization. Scale bars, 50 μm. (ii) Four FOVs in 4 PBS-treated and 4 RAW264.7 macrophage-treated tumor samples scored blindly for disorganization. Data represent means ± SD; significance was determined using unpaired t tests (^∗∗∗^p < 0.001). (D) (i) Timeline depicting clodronate-containing liposome protocol. (ii) Tumor sections from clodronate-treated mice reconstituted with PBS, BMM, or BMM stained with Lyve1-Cy3 or podoplanin-AF555 (red). Lymphatic disorganization within tumors from 6 mice was quantified from >3 FOVs per mouse from Lyve1-stained sections. Data represent means ± SD; significance was determined using unpaired t tests (^∗∗^p < 0.01). (E) Primary LECs were cultured alone, with BMM, eBMM, or TAM. LECs were stained with podoplanin-AF555, and macrophages were stained with F4/80-FITC. Confocal microscopy (x40 air objective) was used to quantify the area of LECs from 3 FOVs (n = 2). Scale bar, 10 μm. (F) (i and ii) Monolayer of SV-LECs (CellTracker Green CMFDA) with RAW264.7 macrophages (CellTracker Orange CMTMR) after 24 h. Area of SV-LECs was measured using ImageJ software. Data represent means ± SEM; significance was determined using unpaired t tests (^∗∗^p < 0.01). Scale bars, 25 μm. (G) (i) SV-LECs transfected with RhoA RAICHU biosensor (RAICHU R/G) or RhoA-GFP as a control. Transfected SV-LECs were cultured alone or with BMM or eBMM for 24 h. (ii) Multiphoton microscopy was used to determine the fluorescence lifetime decay (Tau; in nanoseconds) of SV-LECs transfected with RhoA-GFP or RhoA RAICHU biosensor. Data represent means ± SD; significance was determined using unpaired t tests (^∗∗^p < 0.01). N.S., not significant.

We used a mammary image window (MIW) subcutaneously implanted over a 4T1.2-mCherry tumor (Kedrin et al., 2008; Figure 2A). Injection of 76 kDa dextran-FITC (fluorescein isothiocyanate) allowed visualization of lymphatic vasculature. Using multiphoton microscopy, we observed that, within the tumor, lymphatic vessels leaked dextran dye across the FOV (Figure 2Aii, left panel), suggesting high levels of vessel permeability; however, in more distal regions, lymphatic vessels had a distinct structure and 4T1.2-mCherry intra-lymphatic tumor cells could be seen within vessels, suggesting ongoing metastasis (Figure 2Aii, middle and right panels, respectively). To understand how increasing TAMs could phenotypically influence lymphatic vasculature, we studied the permeability of lymphatic vessels from 4T1.2 tumor-bearing mice given an intermittent bolus of RAW264.7 macrophages during tumor development. Both RAW264.7 macrophages and the 4T1.2 tumor line are derived from a BALB/c genetic background, allowing us to investigate the effects of elevated macrophage numbers on tumor progression *in vivo* using a syngeneic model of TNBC.

To quantify lymphatic vessel permeability *in vivo*, we adapted a protocol previously used in angiogenesis studies (Finsterbusch et al., 2014). Using a subcutaneous injection of Evans Blue dye, we quantified the permeability of the tumoral lymphatics. Tumors with elevated macrophages contained hyperpermeable lymphatic vessels with an increase in mean optical density (OD) per gram from 0.7812 ± 0.2956 to 2.290 ± 0.5160 when compared with PBS-treated control, suggesting a facilitated pathway between the primary tumor and lymphatic vasculature (Figure 2B).

To understand the effects of elevated macrophages on tumoral lymphatic vessel architecture, we stained tumor sections from mice treated with PBS or RAW264.7 macrophages with the lymphatic vessel markers, Lyve1 and podoplanin (Figure 2C; Figures S1A and S1B), demonstrating that both lymphatic markers gave a similar staining distribution. Typical sections from PBS-treated mice showed small, well-formed vessels toward the tumor periphery or within the peri-tumoral areas with a mean diameter of 13.66 μm ± 1.295 μm. This was in contrast to RAW264.7-treated mice that had larger vessels with a mean diameter of 48.00 μm ± 6.065 μm, indicating increased vessel dilation (Figure S1C).

To quantify changes in lymphatic architecture in tumors with elevated levels of macrophages, we blindly scored lymphovasculature for disorganization based on the following criteria. Smaller vessels with a clear lumen were given low scores (0 and 1) compared with larger disorganized vessels with unclear borders (2 and 3). PBS-treated tumors had a mean disorganization score of 0.25 ± 0.16 and 1.6 ± 0.33, compared with 1.8 ± 0.29 and 2.5 ± 0.17 for tumors treated with RAW264.7 macrophages (Figure 2C).

To further investigate whether macrophages were sufficient to induce a disorganized lymphatic phenotype, we ablated endogenous macrophages using clodronate-containing liposomes post-establishment of 4T1.2 tumors. Endogenous macrophages were reconstituted post-clodronate treatment with non-educated bone marrow macrophages (BMMs) or tumor-educated BMMs for 48 h (Figure 2Di). The extent of lymphatic disorganization in the 4T1.2 primary tumors was greater after reconstitution with endogenous tumor-educated BMMs, compared with non-educated BMMs (0.333 ± 0.3 to 2 ± 0.29; Figure 2D, ii and iii). These results demonstrate that the presence of TAMs results in a disorganized lymphatic vasculature around the primary tumor, that the extent of disorganization is related to overall macrophage levels, and that this occurs at an early time point in tumor development (days 10–14).

To investigate how TAMs affect lymphatic endothelia, we added endogenous macrophages to monolayers of primary LECs isolated from BALB/c mice (Figure 2E). Primary LECs had a mean spread area of 1,132 μm^2^ ± 247.9 μm^2^, which reduced slightly to 808.6 μm^2^ ± 185.9 μm^2^ after the addition of endogenous uneducated macrophages but dramatically reduced to 324.1 μm^2^ ± 76.43 μm^2^ with tumor-educated macrophages and 473.7 μm^2^ ± 92.8 μm^2^ with *ex vivo* TAMs (CD45^+^Ly6G^−^CD31^−^CD11b^+^). Similar LEC contraction occurred when the murine LEC line, SV-LEC (Ando et al., 2005), was grown as a monolayer and endogenous macrophages (Figure S1D) or RAW264.7 macrophages added (Figure 2Fi). SV-LEC contraction occurred with areas reducing from 835.9 μm^2^ ± 72.32 μm^2^ to 380.5 μm^2^ ± 40.82 μm^2^ and from 632.5 μm^2^ ± 83.0 μm^2^ to 82.67 μm^2^ ± 14.38 μm^2^. In addition, the area of SV-LECs was quantified with and without contact with RAW264.7 macrophages. SV-LEC contraction was only observed when direct contact between the 2 cell types occurred (436.4 μm^2^ ± 63.3 μm^2^ to 116.2 μm^2^ ± 34.6 μm^2^) (Figure 2Fii). Collectively, our evidence suggests that direct contact between TAMs and LECs is required for contraction events to occur.

RhoA regulates many events in blood-vessel-specific endothelial cells during angiogenesis, such as motility, proliferation, and permeability (Bryan et al., 2010). We sought to test whether RhoA regulates contraction events observed in LECs. SV-LECs were transiently transfected with the GFP- and monomeric red fluorescent protein (mRFP)-expressing RhoA RAICHU biosensor (Heasman et al., 2010, Makrogianneli et al., 2009, Yoshizaki et al., 2003), which allows measurement of the fluorescent lifetime decay (Tau) when fluorescence resonance energy transfer (FRET) occurs between the GFP and mRFP upon RhoA activation. After SV-LEC transfection, non-educated or tumor-educated endogenous macrophages were added to SV-LECs for 24 h. The fluorescence lifetime of the RAICHU probe (expressed exclusively in the SV-LECs) was measured using multiphoton microscopy. SV-LEC co-culture with tumor-educated macrophages led to a reduction in Tau of the biosensor from 1.797 ns ± 0.0252 ns to 1.622 ns ± 0.0338 ns, indicating an increase in FRET between the GFP- and RFP-terminal fluorophores and, consequently, an increase in RhoA activity (Figure 2G). No change in Tau was observed when SV-LECs were co-cultured with non-educated endogenous macrophages (Figure 2Gii). These results demonstrate that RhoA activity increases during LEC contraction and that this only occurs in the presence of tumor-educated macrophages in contact with lymphatic endothelia.

### LEC Contraction Is Dependent on TGF-β1 Release from Tumor-Educated Macrophages

**Figure 3:**
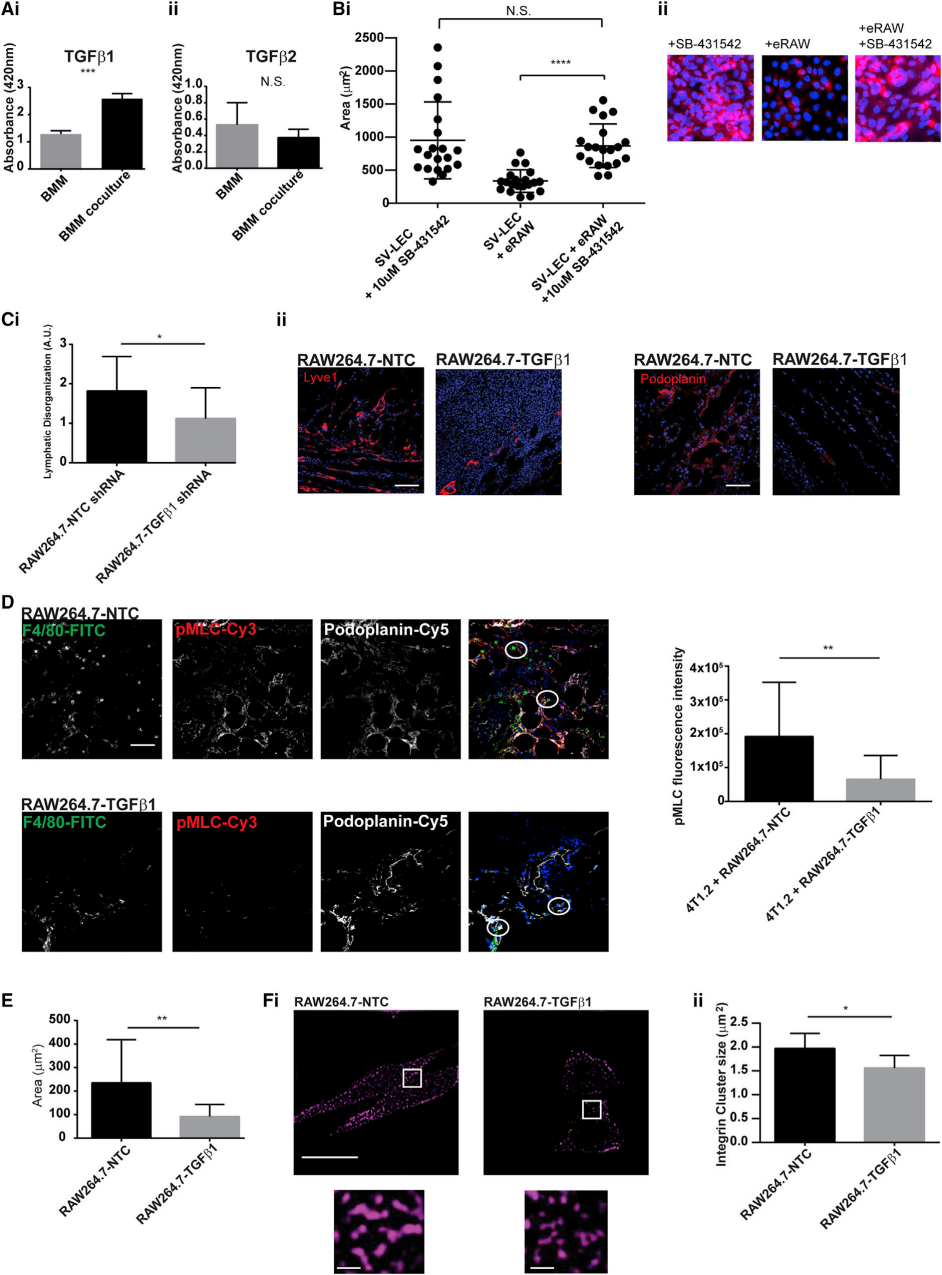
Macrophage-Expressed TGF-β1 Regulates β4 Integrin Clustering on the Macrophage Plasma Membrane and Is Required for LEC Contraction (A) BMMs cultured alone (BMM) or with 4T1.2 cells (BMM coculture). Supernatants were probed for (i) TGFβ1 and (ii) TGF-β2 by ELISA. Data represent means ± SD; significance was determined using unpaired t tests (^∗∗∗^p < 0.001). N.S., not significant. (B) SV-LECs grown as monolayers. Tumor-educated RAW264.7 macrophages (eRAW) were added plus DMSO control or 10 μM SB-431542. After 24 h, SV-LEC areas were quantified. Data represent means ± SD; significance was determined using unpaired t tests (^∗∗∗∗^p < 0.0001). N.S., not significant. (C) Tumor-bearing mice were injected with RAW264.7-NTC or RAW264.7-TGFβ1 knockdown until day 14. Tumor sections were stained with podoplainin-AF555 or Lyve1-Cy3 and Lyve1+ vessels blindly scored for lymphatic disorganization (^∗^p < 0.05). Scale bars, 50 μm. (D) Tumor-bearing mice were injected with RAW264.7-NTC or RAW264.7-TGFβ1 until day 21. Tumor sections were stained with F4/80-FITC, pMLC (and Rabbit-Cy3 secondary antibody), and podoplanin-Cy5. F4/80+ cells within podoplanin+ regions were identified, and a 65-μm^2^ region of interest (ROI) was identified (white circles) where the fluorescence intensity of the pMLC signal was quantified. Scale bar, 50 μm (4 FOVs from n = 2 tumors from each condition). Data represent means ± SD; significance was determined using unpaired t tests (^∗∗^p < 0.01). (E) RAW264.7-NTC and RAW264.7-TGFβ1 macrophage areas were measured by confocal microscopy. Data represent means ± SD; significance was determined using unpaired t tests (^∗∗^p < 0.01). (F) RAW264.7-NTC and RAW264.7-TGFβ1 were stained with anti-β4 integrin-AF647 and imaged using structured illumination microscopy (Nikon ×100 oil objective). Focal adhesion area was determined using ImageJ on thresholded images. Data represent means ± SD; significance was determined using unpaired t tests (^∗∗^p < 0.01). Scale bars, 10 μm (main image) and 1 μm (insets).

Transforming growth factor (TGF)-β receptor ligation in fibroblasts results in RhoA activation (Fleming et al., 2009). We investigated the release of active TGF-β1 and TGF-β2 isoforms from non-educated and tumor-educated macrophages by ELISA (Figure 3A). TGF-β1 levels increased from 2,600 pg to 4,400 pg in tumor-educated endogenous macrophages (increase in optical absorbance at 450 nm from 1.286 ± 0.07119 to 2.585 ± 0.1077). In contrast, TGF-β2 levels were not significantly changed. While TGF-β is present throughout the tumor microenvironment, membrane-bound TGF-β can have a potent effect on downstream signaling through increasing the concentration gradient of this molecule (Savage et al., 2008). Our data showed that 4T1.2 education of endogenous macrophages significantly increased the levels of plasma-membrane-bound TGF-β1 (Figure S2A), allowing stringent spatial control of downstream signaling events.

To test the hypothesis that macrophage-released TGF-β1 was responsible for LEC contraction, we investigated the effect of a TGF-β receptor inhibitor, SB-431542 (Inman et al., 2002; Figure S2B). As expected, RAW264.7 macrophages alone induced LEC contraction (950.6 μm^2^ ± 129.9 μm^2^ to 335.8 μm^2^ ± 38.23 μm^2^); however, this did not occur in the presence of SB-431542 or when TGF-β1 or β4 integrin were transiently knocked down in RAW264.7 macrophages, demonstrating that the presence of β4 integrin and TGF-β in macrophages or TGF-β receptor ligation on LECs was sufficient to prevent contraction (Figures 3B, S2C, and S2D).

The role of macrophage-released TGF-β1 on lymphovascular disorganization was investigated *in vivo*. A stable knockdown of TGF-β1 was generated in RAW264.7 macrophages using lentiviral short hairpin RNA (shRNA) (Figure S2E). Similar to our previous *in vivo* studies, macrophages were administered intravenously throughout tumor development. After 2 weeks’ growth, tissue sections were stained for Lyve1 and podoplanin. The extent of lymphatic disorganization in tumors with RAW264.7-TGFβ1 knockdown, compared with that in RAW264.7-NTC, was blindly scored in Lyve1-podoplanin-stained tissues as described earlier. Our results show that absence of TGF-β1 in RAW264.7 macrophages was sufficient to significantly decrease the extent of lymphatic disorganization observed, compared with that in RAW264.7-NTC macrophages (1.8 ± 0.16 to 1.1 ± 0.18) (Figure 3C) and that these changes were evident at an early time point.

To functionally associate macrophage-released TGF-β1 to structural changes in the lymphatic endothelium *in vivo*, we quantified levels of phospho-myosin light chain (pMLC) in LECs adjacent to macrophages. Since RhoA activity is high in contracting LECs, and since active RhoA phosphorylates MLC, pMLC can be used as a readout of LEC contractility in cells proximal to lymphatic-associated macrophages. We observed that, when mice were injected with RAW264.7-TGFβ1 knockdown, compared with RAW264.7-NTC, there was a significant reduction in pMLC levels in lymphatic vasculature adjacent to RAW264.7 macrophages when TGF-β1 was absent (1.97 × 10^6^ ± 401,151 to 6.56 × 10^5^ × 187,133) (Figure 3D).

### TGF-β1 Controls β4 Clustering at the Macrophage Plasma Membrane

We studied the effect of TGF-β1 on the phenotypic functionality of macrophages by quantifying the spreading response of macrophages. There was clear reduction in cell spreading when TGF-β1 was knocked down in RAW264.7 macrophages, compared with the non-targeted control counterpart (235.2 μm^2^ ± 41.06 μm^2^ to 91.91 μm^2^ ± 11.62 μm^2^) (Figure 3E). To understand how TGF-β1 could control macrophage spreading, we investigated the effect of TGF-β1 on β4 expression. Since integrins can be constitutively expressed on the cell surface, we sought to study the plasma membrane distribution of β4 integrin using structured illumination microscopy in RAW264.7-TGFb1 shRNA versus RAW264.7-NTC. Our results clearly show that, while there may be small differences in the overall amount of β4 integrin expressed on the cell surface (Figures S3A and S3B), the size of integrin clusters that can form firm adhesive contact with integrin ligand are significantly reduced when TGF-β1 is absent (1.97 μm^2^ ± 0.12 μm^2^ to 1.559 μm^2^ ± 0.0.07 μm^2^; Figure 3F, i and ii). These results collectively indicate that TGF-β1 has both a paracrine role in controlling the lymphatic endothelium and an autocrine role in regulating β4 activity in tumor-educated macrophages.

### β4 Integrin+ Macrophages and Lymphatic Remodeling Are Associated with TGF-β Signaling and Adverse Outcome in TNBC Patients

**Figure 4:**
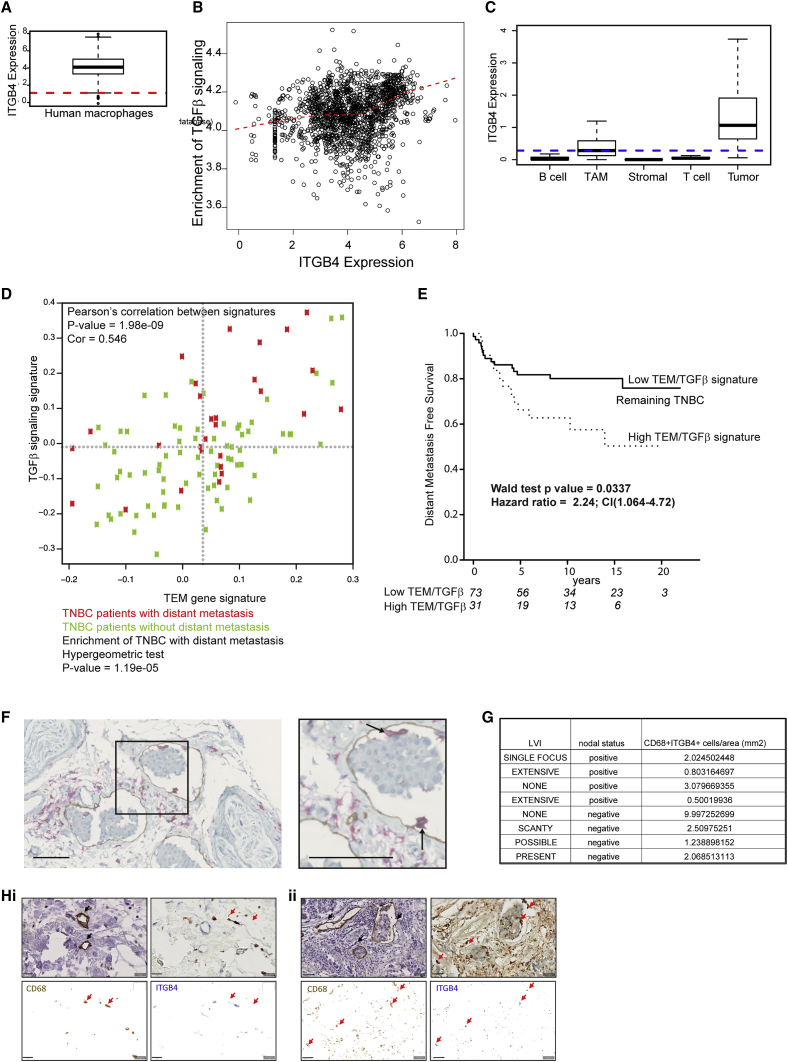
β4 Integrin-Expressing Macrophages and Lymphatic Remodeling Associated with TGF-β Signaling and Adverse Outcome in TNBC Patients (A) ITGB4 expression in human macrophages. The y axis indicates normalized expression on log_2_ scale. Red line indicates median expression of all genes. Raw gene counts were obtained from the ARCHS4 database. (B) Correlation between ITGB4 expression and enrichment of TGF-β signaling in human macrophages (Spearman rho = 0.26; p < 0.001. The x axis indicates normalized expression on the log_2_ scale. The y axis indicates single sample gene set enrichment analysis (ssGSEA) enrichment scores computed for the TGF-β hallmark gene set obtained from the molecular signatures database (MSigDB). Red curve indicates loess fit. Association strength was quantified using Spearman correlation coefficient. Raw gene counts were obtained from the ARCHS4 database. (C) Expression of ITGB4 in single cell RNA sequencing (scRNaseq) data of primary breast cancer (GEO: GSE75688). Data are reported as log_2_(TPM+1). TPM, transcripts per million. (D) Activation score of TEM gene signature and TGF-β signaling. Red and green dots indicate TNBC with or without distant metastasis, respectively. Enrichment of TNBC with distant metastasis in the top right quadrant, established by hypergeometric testing. (E) Kaplan-Meier survival curves showing distant metastasis-free survival in TNBC. Stratification based on samples with high TGF-β signaling and TEM gene signature activation score classified as “High TEM-TGFβ signature” versus the remainder (“Low TEM-TGFβ signature”). (F) Representative breast cancer section (from n = 20) stained with CD14 (red) and podoplanin (brown). Scale bars, 100 μm. Zoomed inset demonstrates CD14+ macrophages associated with podoplanin+ lymphatic vasculature (black arrows). Tissues were selected from 8 patients with or without lymph node positivity. Consecutive sections were stained singly for podoplanin lymphovasculature or doubly using pan-macrophage marker, CD68, and anti-b4 integrin antibody. (G) Double-stained macrophages per square millimeter shown with patient clinical details (LVI and lymph node positivity). (H) CD68+ITGB4+ macrophages are indicated in upper right panels (red arrows). CD68 and ITGB4 stainings are indicated below as 2 single panels; CD68+ITGB4+ macrophages are indicated with red arrows. Podoplanin+ vessels shown in upper left images (black arrows). Scale bars, 20 μm.

To establish that human macrophages express ITGB4 RNA (β4 integrin), we performed an analysis of a compendium of data composed of macrophages from *in vitro* and *in vivo* datasets. We observed that ITGB4 is expressed in both human and mouse total macrophages (Figures 4A and S4A). From the same compendium, a correlation between ITGB4 expression and signaling downstream of TGF-β1 was established (Figure 4B). Single-cell transcriptome analysis of non-tumor cells isolated from primary breast tumors revealed that TAMs expressed high levels of ITGB4, compared with other non-tumor cells within the tumor microenvironment (Figure 4C). To identify patients who may have enrichment of macrophages capable of lymphovascular remodeling, we used a gene signature containing genes enriched in TEMs (Pucci et al., 2009) in a cohort of 122 TNBC gene expression patterns (Gazinska et al., 2013). We plotted the activation score of the TEM gene signature against the TGF-β signaling pathway for each tumor and observed the enrichment of patients with distant metastasis when both of these gene signatures were present in the primary tumor (Figure 4D). Kaplan-Meier plots also showed a significant reduction in distant metastasis-free survival (DMFS) in patients classified as having a high TEM-TGF-β activation score (Figure 4E). To investigate the presence of lymphatic-associated macrophages in breast cancer patients, samples from 20 patients were used. Of these patients, 10 were previously characterized as having lymphatic vessel invasion (LVI), and the remaining 10 did not have LVI. To assess macrophage localization with respect to lymphatic vasculature, we dual-stained sections with an antibody against CD14 and podoplanin (Figure 4F). The sections were scored for the presence of CD14+ macrophages within or proximal to lymphatic vasculature. In our cohort of 20 patients, all samples exhibited some degree of CD14 and podoplanin positivity. Six cases (30%) had macrophages associated with lymphatic vessels; of these, 4 were shown to be positive for LVI. In this small study, our results suggest that 67% of patients with lymphatic-associated macrophages also have LVI. In a separate small patient cohort (8 patients), we demonstrated CD68+ macrophages expressing β4 integrin (ITGB4) in close proximity to podoplanin+ vessels using consecutive paraffin-embedded sections (Figures 4G and 4H). We quantified CD68+ITGB4+ macrophages per square millimeter and saw an association between CD68+ITGB4+ macrophage score and lymph node positivity in individual patients (Figure S4B). Future studies will endeavor to repeat this small study in a larger patient cohort to investigate whether this relationship is statistically significant. The combination of our data suggests that β4-integrin-expressing lymphovascular macrophages may be driving LVI and subsequent metastasis to lymph nodes via the lymphatic remodeling signaling cascade.

## Discussion

This study demonstrates how crosstalk between a previously unreported tumor-infiltrating myeloid subpopulation and an existing lymphatic vasculature can promote metastasis through quantifiable architectural changes in lymphatic vessels. We identified a population of β4 integrin-expressing macrophages that drive lymphatic remodeling through TGF-β signaling and are associated with adverse pathological response in TNBC patients.

Our study uses both endogenous BMMs and the RAW264.7 macrophage cell line, which is strain-matched to the lymphotropic tumor cell line, 4T1.2. Through intravital imaging and *ex vivo* tissue analysis, our TNBC model allowed us to probe the relationship between the tumor lymphatic vasculature and macrophages *in vivo* and directly translate these phenotypic observations into *in vitro* assays for mechanistic studies. We then directly assessed the prognostic significance of the key molecules in the lymphatic signaling cascade in predicting adverse pathological outcome for a cohort of TNBC patients. In breast cancer samples previously characterized for LVI, we identified lymphatic-associated macrophages in approximately a third of the samples and show that LVI was present in the majority of these cases. We identified β4 integrin-expressing macrophages proximal to lymphatic endothelium in breast cancer samples and demonstrate that, in patients with a larger α6β4-expressing macrophage infiltrate, there is a trend toward sentinel lymph node metastasis. Our data suggest that β4 integrin-expressing macrophages may drive metastasis via the lymphovascular route in human breast cancer.

Our study reveals that macrophages are retained in lymphatic endothelium in a TNBC model through the upregulation of β4 integrin on tumor-educated macrophages. While the adhesion receptor α6β4 integrin is ubiquitously expressed in early breast cancer (Diaz et al., 2005), transcriptome analysis of breast cancer patient samples revealed a correlation between expression levels and prognosis (Lu et al., 2008). Through analysis of β4 integrin at the transcriptome and protein levels, we demonstrate a population of endogenous macrophages that express β4 integrin and are adherent to laminin-5 in lymphovascular areas. Collectively, our data suggest that β4 integrin acts to ensure that tumor-infiltrating macrophages are in a prime location for sustained interaction with LECs.

We have defined dual functionality of TGF-β1 where it can affect signaling within TAMs and LECs. First, we show that TGF-β1 is required for β4 integrin clustering at the macrophage plasma membrane. Integrin clustering can positively regulate levels of cell adhesion rapidly in response to soluble stimuli (Hynes, 2002). TGF-β has previously been demonstrated to control α6β1 and α6β4 integrin clustering in HER2-overexpressing mammary tumor cells (Wang et al., 2009). Here, we describe TGF-β1-dependent β4 integrin clustering in macrophages that control the macrophage-spreading response necessary for TAM adhesion at the site of lymphatic vasculature.

Second, TGF-β1 acts in a paracrine manner to activate RhoA in LECs lining the lymphatic vessel, as demonstrated through RAICHU-fluorecent lifetime imaging microscopy (FLIM) technology (Heasman et al., 2010, Makrogianneli et al., 2009, Vega et al., 2011). Our study shows that signaling within LECs in contact with TAMs drives LEC contraction, which correlates to gross architectural changes and hyperpermeability of the lymphatic vessel network that could actively facilitate metastasis. We have previously demonstrated the activation of RhoGTPases by integrin signaling *in cis* (on the immune cells that are triggered by adhesion processes (Makrogianneli et al., 2009, Carlin et al., 2011, Heasman et al., 2010, Ramsay et al., 2013). Our present study indicates that this phenomenon can also occur *in trans*, i.e., activation of RhoGTPases in the endothelial cells that are contacted by the adherent macrophages, through the expression of factors such as TGF-β1. The role of macrophage-released TGF-β1 *in vivo* is shown to have an effect on the RhoA pathway in proximal LECs and a concomitant role in lymphovasculature disorganization.

In summary, this study identifies an alternative macrophage-mediated signaling pathway involved in the promotion of lymphatic metastasis. Our work emphasizes the importance in considering crosstalk between macrophages and the lymphatic vessel network in TNBC, where aggressive tumor growth and rapid metastasis often mean a poor outcome. We hope this study will guide future endeavors to focus on therapeutically targeting the lymphatic remodeling signaling cascade in TNBC disease progression.

## STAR★Methods

### Key Resources Table

**Table:** 

REAGENT or RESOURCE	SOURCE	IDENTIFIER
**Antibodies**		

Rat monoclonal anti-Lyve1	Novus Biologicals	#NB-600-1008
Rabbit polyclonal anti-Tie2 (C-20)	Santa Cruz	#sc-324
Rabbit polyclonal phospho-Smad2/3 (D27F4)	Cell Signaling	#8828
Mouse monoclonal anti-ITGB4	Abcam	#ab29042
Mouse monoclonal anti-CD68 antibody	Ventana Cell Marque	#168M
Mouse monoclonal anti-CD14 (EPR3653)	Ventana Cell Marque	#114R
Mouse monoclonal anti-podoplanin (D2-40)	Ventana Cell Marque	#332M
Rat monoclonal anti-CD45-APC-Cy7	Biolegend	#103115
Rat monoclonal Ly6G-Biotin	Biolegend	#127603
Streptavidin AF488	Biolegend	#405235
Rat monoclonal CD11b-eFluor450	ThermoFisher Scientific	#48-0112-82
Rat monoclonal Tie-2 PE	Biolegend	#124007
Rat monoclonal β4 integrin-BV711	BDBiosciences	#744154
CD31 PerCPCy5.5	Biolegend	#102419
Rat monoclonal anti-F4/80-FITC (clone BM8)	Abcam	#Ab60348
Rabbit polyclonal anti-laminin-5	Abcam	#Ab14509
Rabbit polycloncal Anti-Phospho myson light chain (Ser19)	Cell Signaling	#3671
Mouse monoclonal anti-podoplanin antibody	Santa Cruz	#sc-166906
Rabbit polyclonal anti-TGFb1 antibody	Proteintech	#11522-1-AP

**Biological Samples**		

Breast cancer tumor tissues (paraffin-embedded)	King’s College London breast cancer biobank	Team lead – Dr Cheryl Gillet
4T1.2 tumor tissues (frozen)	King’s College London	Dr Rachel Evans
BLG-Cre;Brca1^f/f^,p53^+/−^ tumor tissues (frozen)	King’s College London	Dr Rebecca Marlow

**Chemicals, Peptides, and Recombinant Proteins**		

Cell tracker™ red (CMTMR) and Cell tracker™ green (CMFDA)	Life Technologies	#C34552, C2925
Murine CSF1	Sigma	#M9170
Human recombinant laminin-5	Novus Biologicals	#H00003911
Clodronate and PBS liposomes	Liposoma Technology	#CP-005-005
2′,7′-bis-(2-carboxyethyl)-5-(and-6)-carboxyfluorescein-acetoxymethyl ester (BCECF)	Thermo Scientific	#B1170
SB-431542	Sigma	#S4317
Evans Blue dye	Sigma	#E2129
Formamide	Sigma	#F9037
76kDa dextran Texas Red	Sigma	#R05027
76kDa dextran fluorescein	Santa Cruz	#sc-263323

**Critical Commercial Assays**		

Murine TGFb1 quantikine ELISA kit	R&D Ltd	#MB100B
Murine TGFb2 quantikine ELISA kit	R&D Ltd	#DB250

**Deposited Data**		

Experiment ArrayExpress accession	Array Express	ArrayExpress: E-MTAB-4064.
Breast Cancer Gene Expression data	Gene Expression Omnibus	GEO: GSE75688
ARCHS4 database	(Lachmann et al., 2018)	N/A

**Experimental Models: Cell Lines**		

4T1.2 cells derived from female BALB/C mouse	(Lelekakis et al., 1999)	N/A
SV-LEC (derived from male “immortomouse”)	(Ando et al., 2005)	Gift from Dr Steven Alexander
Primary LEC from male BALB/C mouse	Generon Ltd	#BALB5064L
RAW264.7 derived from male BALB/C mouse	ATCC Ltd	#ATCC-TIB71
HEK293T (derived from human fetus)	ATCC Ltd	#ATCC-CRL-11268

**Experimental Models: Organisms/Strains**		

Female BALB/c mice	Charles River	N/A
Female C57Bl6J mice	Charles River	N/A

**Oligonucleotides**		

RAICHU RhoA biosensor construct	King’s College London	(Heasman et al., 2010)
GFP-RhoA construct	King’s College London	(Heasman et al., 2010)

**Software and Algorithms**		

TRI2 https://app.assembla.com/spaces/ATD_TRI/wiki	Gray Laboratories Oxford University and University College London	Dr Paul Barber (Barber et al., 2013)
Prism Software	https://www.graphpad.com/scientific-software/prism/	N/A
ImageJ (Fiji)	https://imagej.nih.gov/ij/	N/A
Colocalization plugin for ImageJ	(within this manuscript)	Dr Fred Festy

**Other**		

TGFb1 shRNA (GIPZ)	Open Biosystems	University College London library
ITGB4 shRNA (GIPZ)	Open Biosystems	University College London
RNA easy minikit	Quiagen	#74104
Live/Dead Yellow dye	Invitrogen	#L34959
Affymetrix Mouse Gene 1.0 ST arrays	Thermo Scientific	#901168

### Contact for reagent and Resource Sharing

Further information and requests for resources and reagents should be directed to the Lead Contact, Tony Ng (tony.ng@kcl.ac.uk).

For a detailed description of the experimental procedures please see Supplemental Information.

### Experimental Model and Subject Details

#### Tissue culture

##### Bone marrow macrophages

Monocytes were isolated from female BALB/c mice femurs and cultured in mCSF-1 for 5 d.

##### Cell lines

All cell lines were tested as mycoplasma negative and authenticated by IDEXX Laboratories Ltd, UK.

#### Tumor-bearing mice

##### 4T1.2

BALB/c immune-competent mice were 6–8 weeks of age and maintained under pathogen-free conditions. Tumors were established by injection of 1x10^6^ 4T1.2 (Lelekakis et al, 1999) cells into the mammary fat pad.

##### BLG-Cre;Brca1^f/f^,p53^+/−^

Mammary tumor chunks (approximately 0.2cm^3^) dissected from BLG-Cre;Brca1^f/f^,p53^+/−^ mice (Molyneux et al., 2010) were transplanted orthotopically into mammary fat pads of recipient 5-week old C57BL6J mice. Tumors were grown for 4-8 weeks before mice were culled and tumor tissues harvested.

#### Human breast cancer samples

Paraffin embedded samples (n = 20) (KHP Cancer Biobank Molecular Taxonomy of Breast Cancer International Consortium (METABRIC) dataset cohort) were used. Ten patients were previously characterized as having lymphatic vessel invasion (LVI) and the remaining 10 did not have LVI. Please see SI for details on staining.

#### Study approval

All experiments were performed in accordance with the local ethical review panel, the UK Home Office Animals Scientific Procedures Act, 1986 and the UKCCCR guidelines.

### Method Details

#### RAW264.7 macrophage treatment

Tumor-bearing mice were injected with 100 μL PBS or 1x10^6^ RAW264.7 macrophages starting on the second day after tumor inoculation and repeated every 2 days until the end of the experiment.

#### Clodronate treatment

Endogenous macrophages were ablated using clodronate-containing liposomes (Weisser et al., 2012).

#### Immunofluorescence

Tissue sections were fixed with 4% paraformaldehyde (PFA), blocked in 5% BSA followed by staining. Hoechst-33342 (0.1 μg/ml) was used for nuclear staining and samples mounted using Mowiol (with DABCO). Image acquisition by confocal microscopy was performed using a Nikon Eclipse Ni-E Upright. Image acquisition was conducted using NIS Elements C software and analyzed using ImageJ software.

#### Image acquisition and analysis for colocalization studies in tissue

Cy3 and AF647 dyes were imaged before and after photobleaching using (x20 0.75NA air objective, Nikon) and a cooled CCD detector (Hamamatsu ORCA-03G, 1024 × 1024) with respective integration time of 100 ms and 1000 ms. Dyes were photobleached using a mode-locked Titanium Sapphire Laser (Coherent, Chameleon Ultra 2) tuned at 730 nm with pulse duration of about 200 fs, a repetition rate of 80 MHz and average laser power on the sample of 30 mW. To measure the relative level of β4 integrin expression within the lymphovasculature compared with the rest of the tissue, we measured average AF647 intensity within lymphovasculature areas (high Cy3 intensity) normalized by the average AF647 intensity outside lymphovasculature areas (low Cy3 intensity).

#### Structured Illumination Microscopy (SIM)

RAW264.7-NTC or RAW264.7-TGFβ1 KD were stained with rat anti-β4 integrin antibody and anti-rat AF647 antibody. Image acquisition by SIM was performed using Nikon N-SIM microscope equipped with a 640nm laser, a Andor iXon Ultra 897 EMCCD camera and a 100x 1.49NA oil immersion objective. Images were analyzed using ImageJ software.

#### Mammary imaging window implantation and intravital microscopy

Mammary Imaging Window (MIW) surgery was performed 10-14 days after tumor innoculation (Kedrin et al., 2008). Images shown are representative of a minimum of 5 independent experiments.For imaging lymphatic vasculature, mice were injected subcutaneously at the tail base with 50 μL 76kDa dextran-fluorescein or dextran-Texas red 15 min prior to imaging. Mice were imaged for a maximum period of 4 h per day using a x20 air objective. All post hoc image processing and image reconstructions were done using ImageJ software.

#### Lymphatic vessel permeability

Tumor-bearing mice were injected subcutaneously at the tail base with 1% Evans Blue dye. After 30 min the mice were culled and the tumors incubated in formamide overnight at 55°C. Optical density of formamide was read at 620nm and quantification of lymphatic permeability was given as OD per g tumor.

#### Adhesion assay

Laminin-5 was plated onto 96 well plates overnight at 4°C and non-specific interactions blocked with BSA. Macrophages (5 × 10^6^/ml) were labeled with 1 μM 2′,7′-bis-(2-carboxyethyl)-5-(and-6)-carboxyfluorescein-acetoxymethyl ester (BCECF) for 30 min at room temperature. 100 μL of cells (1 × 10^6^/ml) were added at 37°C, plates washed, and adhering macrophages quantified using a fluorescence microtiter plate reader.

#### Lymphatic endothelial cell contraction

SV-LEC cells or primary lymphatic endothelial cells were grown as a monolayer. On day 3 LECs and macrophages were stained for 30min at 37°C using 1 μg/ml CMTMR or CMFDA respectively. Macrophages were added to SV-LEC monolayers overnight. Confocal images of the co-culture and the area around individual SV-LECs was calculated using ImageJ software.

#### RhoA biosensor

SV-LECs were transiently transfected with the RAICHU RhoA biosensor (Yoshizaki et al., 2003). The biosensor was modified to express GFP and mRFP (Makrogianneli et al., 2009). Multiphoton time-correlated single photon counting FLIM was performed to quantify RhoA biosensor FRET Fluorescence excitation was provided by a Fianium laser, which generates optical pulses with a duration of 40 ps at a repetition rate of 80 MHz. For the imaging of RAICHU-transfected SV-LECs, multi-photon excitation was employed using a solid-state pumped (8-W Verdi; Coherent), femtosecond self-mode locked Ti:Sapphire (Mira; Coherent) laser system (Peter et al., 2005, Barber et al., 2009). Imaging data comprised of 256 × 256 pixel resolution and 256 time channels. The fluorescence lifetime was calculated as described (Barber et al., 2013).

#### TGFβ1 stable knockdown in RAW264.7 macrophages

Stable TGFβ1 knockdown RAW 264.7 macrophage lines were generated by lentiviral transduction using the pGIPZ system (Open Biosystems). Viral packaging was performed by transiently transfecting HEK293T cells with the pGIPZ shRNA transfer vector and the accessory plasmids pCMV-dR8.91 and pMD2G. Stable cell lines were established using three different shRNA lentiviral vectors. RAW 264.7 macrophages were cultured in puromycin (1 μg/ml) to enable the selection of successfully transduced cells and efficacy of knockdown was assessed by western blotting.

#### FACS analysis

RAW264.7 cell lines (TGFβ1-knockdown or NTC) were stained with a Live-Dead Yellow dye followed by staining with a primary rat anti-β4 integrin antibody and anti-rat AF647-conjugated secondary antibody.

Tumors were disaggregated with Collagenase (Sigma UK) and DNase I (Applichem, UK) before staining with Live-Dead Yellow, CD45-APC Cy7, Ly6G-Biotin + Streptavidin AF488, CD11b-eFluor450, Tie-2 PE β4 integrin-BV711 and CD31 PerCPCy5.5. Cells were fixed with 1% PFA and analyzed in a FACS Canto II (BD Biosciences) cytometer. Data analyzed using FlowJo software (TreeStar Inc., Ashland, OR, USA).

#### Human tissue staining

Sections were stained using anti-CD14/anti-podoplanin using Ventana Benchmark Ultra and Ultra view DAB and Alkaline Phosphatase detection systems. Sections were assessed independently by two histopathologists and scored for CD14+ macrophages within or proximal to lymphatic vasculature.

Alternatively, using consecutive sections the first section was stained with anti-podoplanin and the second section stained with anti-ITGB4 anti-CD68. All sections were stained with DAB+ substrate/chromagen. All incubations were at room temperature.

The slides were scanned in the Hamamatsu NanoZoomer S210 Digital slide scanner. The image analysis was performed on the whole section with the color deconvolution module and the positive pixel algorithm from QuPath image analysis software.

### Quantification and Statistical Analysis

#### Gene expression microarray analysis

RNA was extracted from macrophage cell cultures and profiled using Affymetrix Mouse Gene 1.0 ST arrays. Differential expression between conditions was estimated by fitting a linear model and performing empirical Bayes moderated t tests using the package ‘limma’ (v3.22.4) (Ritchie et al., 2015). The expression score for a specific gene in each sample is defined as the weighted sum of gene-standardized (*Z*-score) expression values, with weights +1/-1 according to relative increase or decrease in BMM + 4T1.2 compared with BMM.

#### Analysis of gene signatures

To establish ITGB4 expression and assess association between ITGB4 expression and activation of the TGFβ signaling in macrophages, processed gene counts were obtained from the ARCHS4 database (Lachmann et al., 2018) and further normalized for downstream analyses. Enrichment of TGFβ signaling was computed using the ssGSEA method (Barbie et al., 2009) as implemented in the GSVA package from Bioconductor.

False zero expression due to dropout events in scRNA-seq data was corrected using the scImpute algorithm as previously described (Li and Li, 2018). scRNaseq data is reported as log2(TPM+1).

Macrophage-mediated vascular remodeling pathway signature (Pucci et al., 2009) was converted to a human gene list using Biomart ID conversion (Ensembl Genes 84// *Mus musculus* genes GRCm38.p4). TGFβ (KEGG) gene signature was derived from (MSigDB). Gene signature activity was calculated using a weighted average sum over all genes for each tumor. Pearson’s correlation between the activation scores was reported. Hypergeometric testing was used to establish the significance of overlap between TNBC with distant metastasis (DM) on those of dual high activation scores. Kaplan-Meier plots were generated for each dataset to provide a visualization of survival stratification.

All other statistical analysis is described in the text and legends and was performed using Prism software (GraphPad). P values less than 0.05 were considered significant. The statistical test used is indicated in the figure legends and the significance of findings is indicated in the figures.

### Data and software availability

The accession number for the microRNA experimental data reported in this paper is ArrayExpress: E-MTAB-4064.

## Supplemental Information

Document S1. Figures S1–S4

Document S2. Article plus Supplemental Information
