# Supplementary material for: Integrin-Mediated Macrophage Adhesion Promotes Lymphovascular Dissemination in Breast Cancer
Source: Cell Rep. 2019 May 14;27(7):1967–1978.e4. doi: 10.1016/j.celrep.2019.04.076 (PMC6527923; doi:10.1016/j.celrep.2019.04.076)
Supplement: Document S1. Figures S1–S4 [file mmc1.pdf]

Cell Reports, Volume *27*

Supplemental Information

Integrin-Mediated Macrophage Adhesion Promotes Lymphovascular Dissemination in Breast Cancer

Rachel Evans, Fabian Flores-Borja, Sina Nassiri, Elena Miranda, Katherine Lawler, Anita Grigoriadis, James Monypenny, Cheryl Gillet, Julie Owen, Peter Gordon, Victoria Male, Anthony Cheung, Farzana Noor, Paul Barber, Rebecca Marlow, Erika Francesch-Domenech, Gilbert Fruhwirth, Mario Squadrito, Borivoj Vojnovic, Andrew Tutt, Frederic Festy, Michele De Palma, and Tony Ng

Figure S1

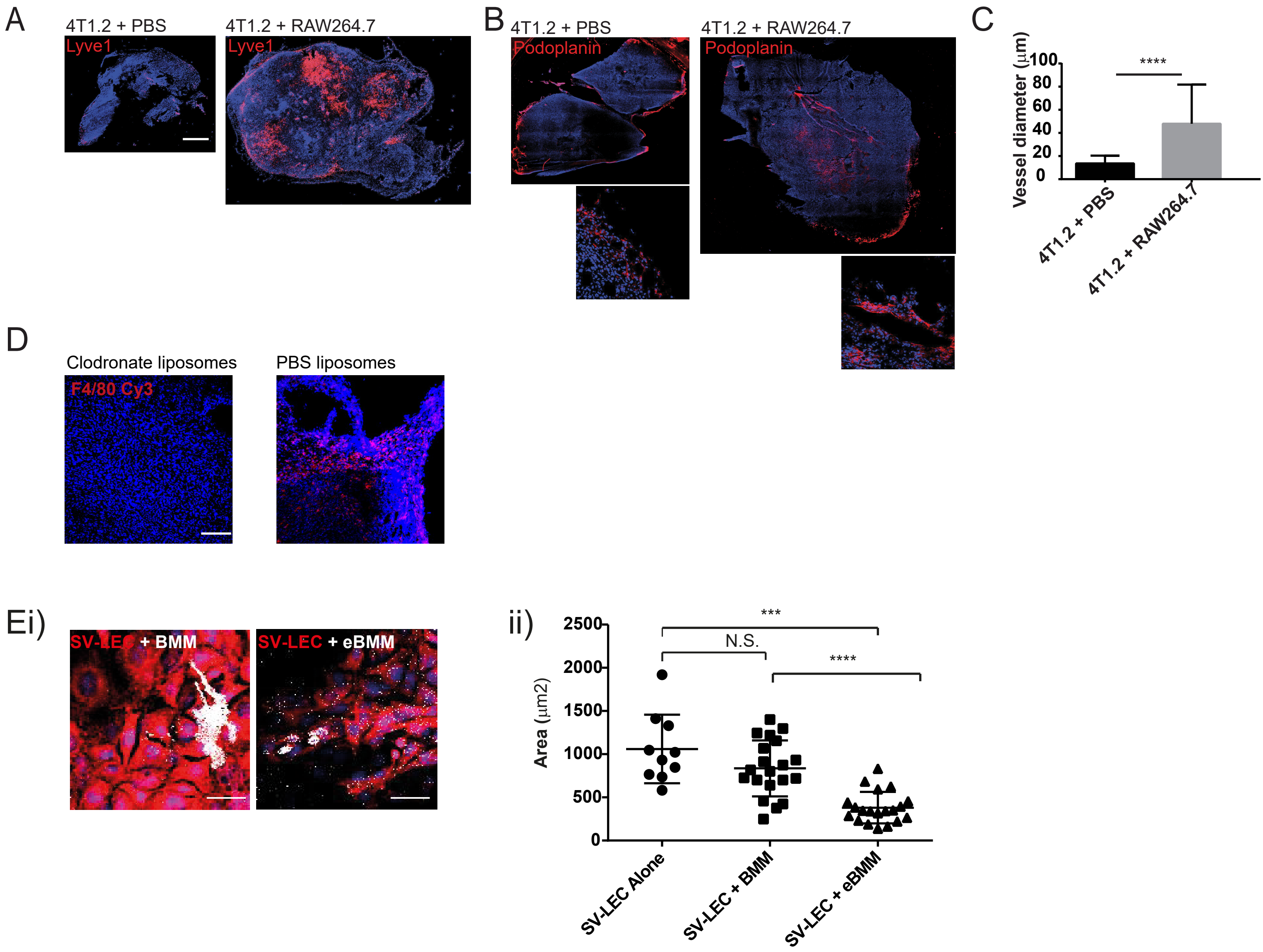

Figure S1. Elevated macrophages in tumor-bearing mice increases lymphatic vessel diameter and contact between SV-LEC and endogenous tumor-educated macrophages results in LEC contraction, related to Figure 2

Tile scans representative of whole tumor sections from mice treated with PBS or RAW264.7 macrophages.10 μm fixed sections were stained with (A) Lyve1 antibody and Cy3-conjugated secondary antibody or (B) podoplanin-AF594 (red) to allow confocal imaging of lymphatic vasculature i) (x4 objective, scale bar, 50 μm) (C) The maximum diameter across Lyve1+ vessels was measured in image J from at least 4 fields of view from each tumor section from 4 PBS-treated and 4 RAW264.7-treated mice. (D) Representative images of tumour tissues from mice treated with PBS liposomes or clodronate liposomes. F4/80-Cy3 (red) depicts infiltrating macrophages (E) SV-LECs were grown as a monolayer on a glass coverslip and stained with CMTMR (red) or CMFDA (green). Non-educated and tumor-educated bone marrow macrophages (BMM and eBMM respectively) were stained with F4/80-Cy5 (white) and both cell types were stained with Hoescht-33342 (blue).

Figure S2

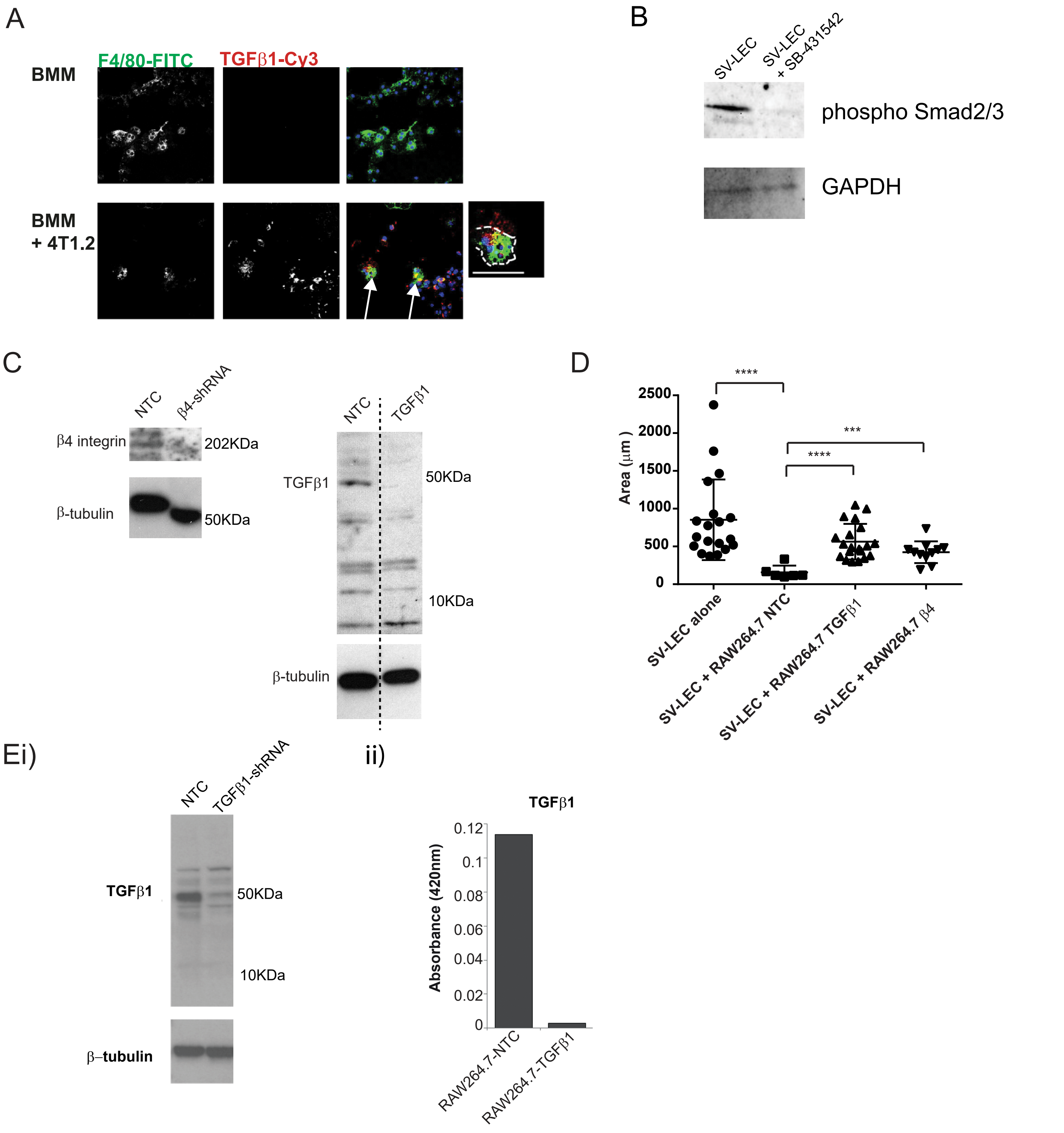

Figure S2. Western blot analysis of TGFβ1 knockdown in RAW264.7 cells, related to Figure 3
(A) Bone marrow macrophages were cocultured alone or with 4T1.2 tumor cells for 5 days on glass coverslips. Cells were fixed with 4% PFA before staining with F4/80-FITC and TGFβ1 and a Cy3 secondary antibody and imaging by confocal microscopy. Cell boundary depicted with white dotted line in inset image. (B) SV-LECs treated with SB-43142 were analysed by western blot to assess levels of phospho Smad2/3 (C) RAW264.7 were transiently transfected with shRNA against β4 integrin or TGFβ1 and analysed by western blot. (D) Macrophages were cocultured with SV-LECs and the contraction of SV-LECs measured (E) RAW264.7 cells were virally transduced with shRNA against TGFβ1 or NTC. (i) Macrophages that were transfected were selected for GFP expression and lysed using an SDS buffer. Lysates were run on a reducing gel (4-12% Bis-Tris), blotted onto PVDF and the membranes were probed for TGFβ1 or β-tubulin as a loading control. (ii) Supernatants were analysed by ELISA for TGFβ1 levels.

Figure S3

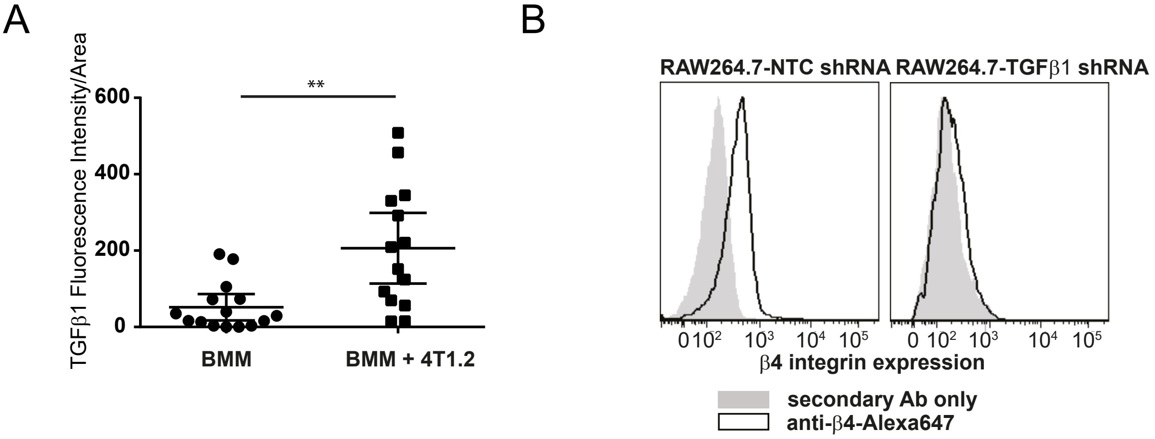

Figure S3. Endogenous macrophages increase expression of membrane-bound TGFβ1 after co-culture with 4T1.2 tumor cell, related to Figure 3

(A) The fluorescence intensity from F4/80+ cells was quantified (white arrows) and normalized to cell area. Scale bar, 10μm. Data represent means ± SD, significance was determined using unpaired t-tests (**$p<0.01$). (B) NTC- and TGFβ1-KD RAW264.7 macrophages were stained with rat anti-β4 integrin followed by secondary goat anti-rat-AF647 antibody. Expression levels of β4 integrin were analyzed by FACS and a representative histogram is depicted (n = 2 independent experiments).

Figure S4

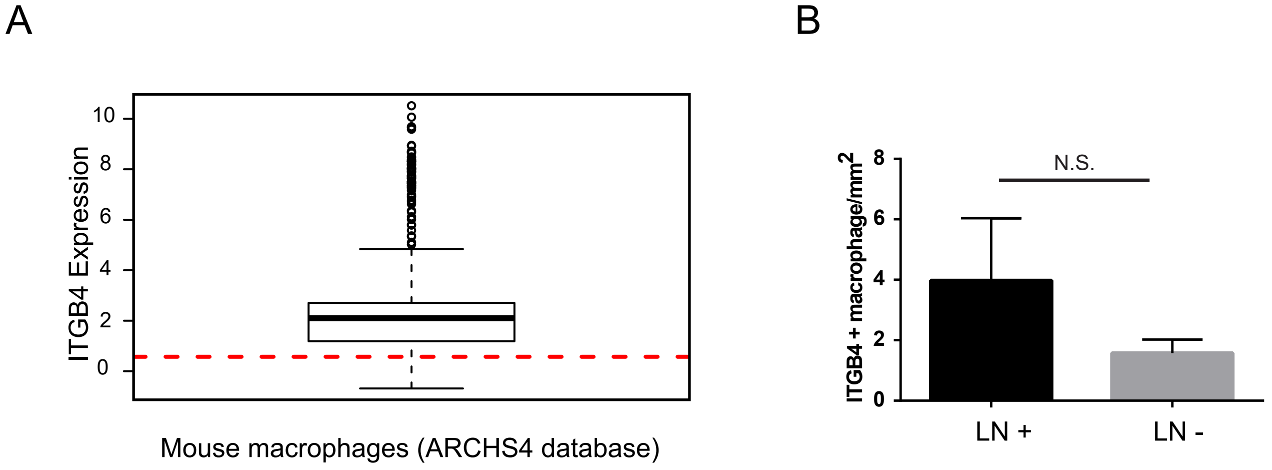

Figure S4. Expression of ITGB4 in murine macrophages (A) ITGB4 expression in murine macrophages, related to Figure 4
The y-axis indicates normalized expression on the log2 scale. The red line indicates median expression of all genes. Raw gene counts were obtained from the ARCHS4 database. (B) CD68+ITGB4+ macrophages in lymph node negative versus lymph node positive tissues are shown graphically.
